# Comparing patterns of sexual risk among adolescent and young women in a mixed-method study in Tanzania: implications for adolescent participation in HIV prevention trials

**DOI:** 10.7448/IAS.17.3.19149

**Published:** 2014-09-08

**Authors:** Elizabeth E Tolley, Sylvia Kaaya, Anna Kaale, Anna Minja, Doreen Bangapi, Happy Kalungura, Jennifer Headley, Joy Noel Baumgartner

**Affiliations:** 1 Social and Behavioral Health Sciences, FHI 360 Durham, NC, USA; 2 Muhimbili University of Health and Allied Services, Dar es Salaam, Tanzania

**Keywords:** adolescents, clinical trials research, sexual and reproductive health, HIV prevention, Tanzania

## Abstract

**Introduction:**

Despite the disproportionate impact of HIV on women, and adolescents in particular, those below age 18 years are underrepresented in HIV prevention trials due to ethical, safety and logistical concerns. This study examined and compared the sexual risk contexts of adolescent women aged 15–17 to young adult women aged 18–21 to determine whether adolescents exhibited similar risk profiles and the implications for their inclusion in future trials.

**Methods:**

We conducted a two-phase, mixed-method study to assess the opportunities and challenges of recruiting and retaining adolescents (aged 15–17) versus young women (18–21) in Tanzania. Phase I, community formative research (CFR), used serial in-depth interviews with 11 adolescent and 12 young adult women from a range of sexual risk contexts in preparation for a mock clinical trial (MCT). For Phase II, 135 HIV-negative, non-pregnant adolescents and young women were enrolled into a six-month MCT to assess and compare differences in sexual and reproductive health (SRH) outcomes, including risky sexual behaviour, incident pregnancy, sexually transmitted infections (STIs), reproductive tract infections (RTIs) and HIV.

**Results:**

In both research phases, adolescents appeared to be at similar, if not higher, risk than their young adult counterparts. Adolescents reported earlier sexual debut, and similar numbers of lifetime partners, pregnancy and STI/RTI rates, yet had lower perceived risk. Married women in the CFR appeared at particular risk but were less represented in the MCT. In addition, adolescents were less likely than their older counterparts to have accessed HIV testing, obtained gynaecological exams or used protective technologies.

**Conclusions:**

Adolescent women under 18 are at risk of multiple negative SRH outcomes and they underuse preventive services. Their access to new technologies such as vaginal microbicides or pre-exposure prophylaxis (PrEP) may similarly be compromised unless greater effort is made to include them in clinical trial research.

## Introduction

In 2010, 34 million people were living with HIV and approximately half of the adult cases were among women [1]. Although estimates of HIV incidence have begun to decline in general populations, young African women aged 15–24 continue to bear a high burden of the disease [1, 2]. Indeed, in Tanzania, HIV prevalence in young women is approximately 3%, three-fold higher than in men of the same age group [3]. As elsewhere, Tanzanian young women's risk of HIV has been attributed to early sexual debut [4] and fuelled by poverty and socio-cultural conditions that undermine the use of currently available HIV prevention methods, such as condoms [5–10] .

Despite the disproportionate impact of HIV on young women, they are markedly underrepresented in clinical trials of new HIV prevention methods. Although a number of potential prevention approaches, including antiretroviral-based vaginal and oral products, have been evaluated in women aged 18 and older, only two trials to date have enrolled women below the age of 18 [11–13] .

There is some recent evidence that younger adults in PrEP trials have poorer product adherence and trial retention, thus undermining our ability to show product effectiveness [14]; however, many have argued that adolescents’ inclusion in clinical microbicide trials is a matter of social justice, providing important opportunities to assess the safety, acceptability and effectiveness of new prevention products within a high-risk population who might differ from their young adult counterparts in important ways [11, 15–17]. The social justice argument may well supersede the logistical needs of trials, or rather, further highlight the special attention and work required to adequately include adolescents in trials. Furthermore, because regulatory bodies such as the United States Food and Drug Administration and the South African Medicines Control Council are unlikely to allow microbicides to be marketed to adolescents without data demonstrating that these products are safe and effective for the adolescent population [18], their exclusion is likely to delay or even limit adolescent access to new HIV prevention products.

Nevertheless, multiple barriers exist to the recruitment of adolescent women, including ethical concerns about younger women's biological safety and their cognitive ability to give informed consent for trial participation, as well as the legal and social challenges of recruiting young, sexually active minors [15, 16, 18]. In addition, although epidemiological research clearly identifies young women as a high-risk group for HIV, little research has been conducted to distinguish the risk behaviours and cultural contexts that put specific adolescents at risk [19]; without more information on adolescent sexual behaviour, it is uncertain whether younger women's patterns of sexual risk behaviours would even make them good candidates for trial participation.

In order to assess the opportunities and challenges of recruiting adolescent women into HIV prevention clinical trials, we conducted a mixed-method study in Dar es Salaam, Tanzania that included community formative research (CFR) and a six-month mock clinical trial (MCT). The specific objectives of the CFR were manifold but included gathering more in-depth information from socio-demographically diverse adolescents and young adult women representative of the types of participants we anticipated being recruited into the MCT in order to finalize study procedures and measures for the MCT. For this paper, we use data from both the CFR and the MCT to identify and compare the sexual relationship patterns, HIV risk perception, HIV risk reduction behaviours and incident sexual and reproductive health (SRH) outcomes of adolescents aged 15–17 with those of young women aged 18–21 to determine whether they face similar or unique risks and to determine how such risks might affect trial participation.

## Methods

This study comprised two research phases – the CFR and the MCT. During the CFR phase, we conducted a total of 64 in-depth interviews (IDI) – for up to three IDIs each with 23 sexually active young women aged 15–21 from different sexual risk contexts: married adolescents, married young women, single in-school and single out-of-school adolescents and young women, of whom some were currently or previously engaged in sex work. [Note: two participants completed only the first IDI and one participant completed the first and second interview, but not the third.] The study team worked with representatives from community-based organizations and schools, community and government leaders and members of a “Youth Interactive Group” (YIG) to identify and recruit participants from 14 very-low-income residential locations with high concentrations of bars, brothels and retail alcohol outlets. Interviews were conducted in Swahili by trained female interviewers, audio-recorded, transcribed and uploaded into NVivo8, a software programme that assists with qualitative data management and analysis. The first and second interviews explored participants’ home life, school or work situation, first and current sexual relationships, reproductive and sexual health knowledge and use of protective behaviours. The third interview focused on their attitudes towards pre-exposure prophylaxis (PrEP), microbicides and participation in HIV prevention clinical research.

During the second research phase, we enrolled 135 sexually active, HIV-negative, non-pregnant adolescents and young women aged 15–21 into a MCT with interviews at baseline, two, four and six months. Research staff conducted informational meetings in communities identified during the CFR and worked with community representatives and YIG members to recruit participants for the MCT. The study was conducted at the Infectious Disease Clinic (IDC) in Dar es Salaam, which offers youth-friendly services. During the study, participants received urine pregnancy tests at each visit and were tested for HIV, bacterial vaginosis (BV) (via gram stain test) and trichomoniasis (via microscopy), at baseline and month four. Those who tested positive were called and offered treatment. They also received behavioural assessments at every visit, with more extensive assessments at baseline and six months.

Qualitative CFR data were analysed thematically following a process of reading, coding and the development of analytic memos and data reduction matrices [20]. Initial codes were proposed and discussed by the US and the Tanzanian research team after reading the first transcripts from interview 1. The codebook was updated after each round and coding took place in parallel to data collection. Memos identified and described dimensions of coded themes (i.e. identifying common contexts within the *HIV Risk Perception* code in which participants did or did not express concern about HIV risk). Important themes/sub-themes were quantified and typed into Excel matrices to identify patterns across age groups and/or risk contexts. For the MCT, we conducted chi-square tests or Fisher's exact tests, as appropriate depending on cell size, to examine bivariate associations of socio-demographic, sexual risk behaviour and SRH risk outcomes. Given our small sample size, we did not further develop multivariable models to assess differences in adolescent versus young women's risk profiles.

Ethical approvals were received from Muhimbili University of Health and Allied Services, the National Institute for Medical Research and FHI 360's Protection of Human Subjects Committee. Participants aged 16 or older gave written informed consent; for 15-year-old participants, a parent/guardian and the participant gave written consent.

## Results

### CFR phase

A total of 23 adolescents and young women participated in the CFR phase. Our analysis of sexual relationships, risk reduction behaviours and risk perception revealed few differences by age group, but many more by risk context (Table 1). More than two-thirds of CFR participants had fractured family lives in which one or both parents had died or were divorced. In many cases – but especially among five young women engaged in sex work – these precarious beginnings appeared to enhance their vulnerability. Each of these young women described a scenario in which the death or divorce of parents and the inability or unwillingness of other relatives to help care for their needs led to their entry into sex work. All but one lived on their own, in a brothel or with a friend. This is in contrast to in-school youth, all of whom were living with one or both parents.

**Table 1:** Community formative phase characteristics by risk group and age

	Sex workers (*N*=5)	Married participants (*N*=8)	Single, out-of-school (*N*=5)	Single, in school (*N*=5)	Adolescents (15–17) (*N*=11)	Young women (18–21) (*N*=12)

	*N* (%)	*N* (%)	*N* (%)	*N* (%)	*N* (%)	*N* (%)
Mean age	17.2	18.4	18.0	18.4	16.4	19.6
Mean years of education	8.4	6.9	8.3	10.2	7.2	9.3
Living with one or both parents	1 (20)	1 (13)	1 (20)	5 (100)	4 (36)	4 (33)
Living with husband or partner	0 (0)	7 (87)	0 (0)	0 (0)	3 (27)	4 (33)
Living with relative	0 (0)	0 (0)	3 (60)	0 (0)	3 (27)	0 (0)
Living on own (brothel/with friend)	4 (80)	0 (0)	1 (20)	0 (0)	1 (9)	4 (33)
Parents separated/divorced	2 (40)	1 (13)	3 (60)	2 (40)	4 (36)	4 (33)
One or both parents deceased	3 (60)	6 (75)	2 (40)	0 (0)	5 (45)	6 (50)
Currently employed	5 (100)	4 (50)	3 (60)	1 (20)	5 (45)	8 (67)
Two or more lifetime sex partners	3 (100)^a^	6 (75)	5 (100)	5 (100)	8 (72)	8 (67)
Ever described partner abuse	0 (0)	7 (87)	2 (40)	0 (0)	4 (36)	5 (42)
Ever tested for HIV	3 (75)^a^	7 (87)	2 (40)	3 (60)	7 (64)	8 (67)
Ever RTI symptoms	2 (40)	3 (37)	2 (40)	3 (60)	2 (18)	8 (67)
Ever pregnant	1 (20)	7 (87)	2 (40)	4 (80)	6 (54)	8 (67)
Currently using condoms	5 (100)	2 (25)	3 (60)	2 (40)	6 (54)	6 (50)
Ever used hormonal contraception	2 (40)	2 (25)	2 (40)	0 (0)	1 (9)	5 (42)

a Some missing information due to lack of second or third interviews.

### Sexual relationship patterns

Sexual debut ranged from 13 to 18 years and was almost two years earlier, on average, for adolescents (14.8 years) than young women (16.6 years). For seven of the eight married participants, the first sexual partner was their husband, although sex often occurred before marriage. Regardless of risk context, first partners tended to be older (3–6 years) than participants, with several husbands older by as much as 11–17 years. No first sexual encounters were reported as physically forced – however, several participants felt they were tricked into having sex the first time.

Participants’ accounts of their current sexual relationships did not appear to vary by age, but did vary by sexual context. Only two of the eight participants who were or had been married described their husbands in positive terms. More commonly, they used terms such as “cruel” or “harsh,” or described husbands as prone to humiliate or insult them. All four of the married adolescents described being controlled and sometimes beaten by their husband. A 17-year-old who was currently staying at her mother's house admitted that “there was too much humiliation … A small mistake and he beats me.”

In contrast, sex workers and in-school youth tended to describe their relationships in more positive terms than the other participants. For example, an 18-year-old sex worker described her boyfriend as “not very capable” of much financial support but able to satisfy her sexually. She added “I am enjoying (sex) to a large extent; that is why we love each other.” A 21-year-old single university student described the emotional and material support she derived from her current partner, a non-Tanzanian businessman: “He is so caring; when I fall sick he takes me to the hospital; when I am hungry he cooks for me. So, he somehow treats me like a baby. … He gives me daily company.”

The word “trust” was often used to describe participants’ current relationships. The term, however, conferred multiple and sometimes contradictory meanings, including emotional and material support, and sexual discretion but rarely sexual exclusivity. A 20-year-old, who reported being married from the age of 13 or 14, was both doubtful and trustful of her husband's behaviour, “I trust my husband; if he has women outside, he will use a condom … fidelity is so important to me, because I trust someone – because he is not a player. He doesn't do dirty things … he is not just having sex with women. That is why I trust him.”

### Risk reduction behaviours

About half of the CFR participants (similar proportions of adolescents and young women) reported currently using condoms, although this varied widely by risk context. Only two of the eight married women used condoms with their husbands and about half of the unmarried in-school and out-of-school participants used them with one or more partners; most described using condoms to avoid pregnancy as well as disease. In contrast, all of the participants engaged in sex work described consistently using condoms, mostly to avoid HIV/STIs.

Adolescents and young adult women suggested that condom use was acceptable or even expected at the beginning of a new relationship. However, most participants described having stopped condom use within their own relationships as “trust” had been established. Several participants suggested that trust was more firmly established after both had tested negative for HIV. The majority of participants who had ever tested for HIV did so within the context of pregnancy or because of illness, although two students and two sex workers described more routine testing behaviour. Five married participants and one out-of-school participant said their partners refused testing.

Among those who were not sex workers, more-consistent condom use appeared to be for the purpose of preventing pregnancy. Only four participants reported currently using a modern, non-barrier method, including two injectable users and one participant each using oral pills and an implant. Several others reported having used or considered using an injectable previously, but stopped or did not initiate use out of fear of side effects.

### Risk perception

Most participants recognized they were at some risk of HIV, but about half appeared reluctant to attribute that risk directly to their own or their partner's behaviours. A 17-year-old married adolescent both trusted and did not trust her 20-year-old husband: “I think I can get HIV if my husband goes outside marriage, if he has sex with a person that is HIV positive, because I don't use protection … I don't use protection because I trust my husband.” To protect herself, she said that it was important “to be settled with my husband and not to have many men. Myself – I can settle, but I don't know about my partner.”

A few participants were more direct in their acknowledgement of risk. A 21-year-old university student admitted that, “Most of us young ladies get HIV because of having relationships for money or other material things, so that drives you to have reckless sex. For example, I can't face somebody and tell him ‘Let's go and test.’ I can't, I will just test myself, so that lack of confidence … leads to acquiring HIV.” Similarly, a sex worker stated, “I have told you my business; worrying is inevitable.” Several participants assessed their risk as minimal, because they took precautions. For example, a 20-year-old university student agreed that she was “of course concerned about AIDS, but not too concerned about other disease … because I protect myself …. Also, (I use) condoms and then I don't mix people. If I have one (partner), it's just him. Maybe if we breakup, that's when I go to another person.”

### SRH outcomes

Participants’ knowledge about sexually transmitted infections (STIs) was variable. Almost half of participants described having ever experienced reproductive tract symptoms, including itching, “fungus,” unusual vaginal discharge or painful intercourse. A few women appeared to use home remedies such as “Dettol” (an antiseptic liquid soap), although most sought treatment from a health facility. However, while participants described being provided medications for treatment, they rarely described being counselled about the source of infection or about ways to reduce sexual risk.

Unprotected sex and unplanned pregnancy were common among the CFR participants. More than half (14 of 23) had ever been pregnant – almost equally divided by age group. Only 7 of the 14 ever-pregnant participants had living children and two adolescent participants became pregnant during the CFR. Four participants, two in each age group, reported having aborted at least once.

### MCT phase

Adolescents and young adult women who participated in our MCT differed from the CFR qualitative sample in several important ways. First, they appeared to come from more stable homes, given that the majority of MCT participants (83% of adolescents and 58% of young adult women) were living with one or both parents. In addition, we enrolled few married women into the MCT. However, there were also striking similarities between the CFR and the MCT phases, especially in terms of sexual debut, patterns of sexual risk and risk reduction behaviours. Below, we describe the MCT participants and patterns of sexual risk, with a focus on whether and/or how adolescents differed from their adult counterparts. Table 2 describes the socio-demographic, sexual risk and protective factors by age group.

**Table 2:** Mock clinical trial: sexual risk and protective factors at baseline, by age group

	Adolescents (aged 15–17) (*N*=41)	Young women (aged 18–21) (*N*=94)	*p*
Age (mean)	16.4	19.3	
Years of education (mean)	8.4	8.9	0.18
Relationship status	%	%	
Married or cohabitating	2.4	9.6	
Regular partner, not cohabitating	97.6	79.8	0.02a
Single	0.0	10.6	
Living with one/both parents	% 82.9	% 58.5	0.006
Occupation	%	%	0.13
Paid employment	22.0	30.9	
Student	29.2	14.9	
Unemployed, not in school	48.8	54.2	
Religion	%	%	
Muslim	56.1	52.1	0.36^a^
Catholic	39.0	34.0	
Other	4.8	13.8	
Mean age at sexual debut	15.2	16.3	
Range	(11–17)	(10–20)	0.0002
First sexual encounter coerced/forced	34.2%	45.7%	0.21
First sexual partner 5+ years older	(*N*=36)	(*N*=87)	1.00^a^
	8.3%	10.3%	
Mean number of lifetime sex partners	1.7	2.6	
Range	(1–5)	(1–10)	0.002
Mean number of sexual partners per year of sexual exposure	1.24	1.06	0.24
Two or more partners in lifetime	39.0%	72.3%	<0.001
Ever concurrent partners	19.5%	16.0%	0.61
Ever exchanged sex for money or gifts	14.6%	13.8%	0.09
Ever tested for HIV	47.5%	75.0%	0.002
Ever had a pelvic exam	5.1%	15.7%	0.14^a^
Ever used modern contraceptives	2.4%	21.3%	0.004^a^
Current contraceptive use (non-condom)	0.0	14.9	0.006^a^
Had sex in last week	(*N*=41) 51.2%	(*N*=93) 49.5%	0.85
Condom at last sex	43.9%	50.0%	0.51
Alcohol at last sex	12.2%	8.5%	0.53^a^
Forced/coerced last sex	4.9%	8.5%	0.72^a^
	(*N*=41)	(*N*=93)	0.005^a^
Perceptions of HIV risk	%	%	
No perceived HIV risk	36.6	15.1	
Perceived a little HIV risk	58.5	64.5	
Perceived a lot of HIV risk	4.9	20.4	
Any incident SRH outcome^b^	(*N*=35) 22.9%	(*N*=69) 14.5%	0.29
Incident pregnancy	17.1%	10.1%	0.31
Diagnosis of bacterial vaginosis or trichomoniasis after baseline^b^	(*N*=22) 9.1%	(*N*=55) 5.5%	0.62^a^

a Fisher's exact test used for any cells with values of 5 or less

b subsample includes only those who had a month 4 clinic visit, during which pelvic and lab tests were conducted. RTI=reproductive tract infection; SRH=sexual and reproductive health; STI=sexually transmitted infection.

### Socio-demographics

Overall, the MCT sample of 135 participants was more similar to the group of single out-of-school CFR participants than to other risk contexts. They had similar years of education and work. About half of the participants were neither in school nor employed. Almost all adolescents (98%) and most young adult women (80%) were single, but had a regular partner.

### Sexual relationship patterns

As in the CFR, adolescents who joined the MCT reported similar or higher risks than their young adult counterparts. On average, adolescents initiated sex about six months earlier than did young adult participants (at 15.2 versus 16.3 years of age.) More than a third of participants, with similar proportions of adolescents and young adult women, reported their first sexual encounter to be coerced or forced. About 10% of participants’ first sexual partners were five or more years older than themselves.

At baseline, adolescents reported fewer sex partners over their lifetime (range, 1–5) than young adult women (range, 1–10). However, mean number of sexual partners was similar when adjusted for years of sexual exposure. About 14% of participants reported ever exchanging sex for money or goods, with no differences by age. There were no statistically significant differences in current sexual or substance use risks across age categories. Half of the participants in each age group had engaged in sex during the week prior to enrolment and about half reported using a condom at last sex. About 10% of participants reported using alcohol during their last sexual encounter; 4.9% of adolescents and 8.5% of adult participants reported that their last sexual encounter was coerced or forced.

### Risk reduction behaviours and risk perceptions

The rate of having ever used contraception was low; no adolescents and fewer than 15% of young women were currently using a modern contraceptive method. Condom use at last sex was similar between age groups. Although the difference was not statistically significant, fewer adolescents (about half) than young women (nearly two-thirds) reported any condom use in the past four weeks (data not shown).

Despite early sexual debut and some similarities in patterns of sexual risk, over a third of adolescents (36.6%) perceived themselves to be at no risk of HIV, compared to 15.1% of their counterparts.

### SRH outcomes

Over the six-month study period, there were 13 incident pregnancies (six in adolescents and seven in young women) among the 104 participants who had at least one follow-up visit and five confirmed cases of incident RTIs/STIs (three cases of BV and two cases of trichomoniasis) among the 78 participants who attended the four-month visit and completed lab tests. In addition, three cases of BV and five cases of trichomoniasis were identified and treated at enrolment. No participant was diagnosed more than once with an RTI/STI. Four young women and one adolescent were not enrolled in the MCT due to a positive HIV diagnosis at screening (data not shown). However, no incident HIV infections were detected post-enrolment. Although not statistically different, almost 20% of adolescents and 10% of young adult women became pregnant during this six-month study.

## Discussion

The findings from this study have several implications when considering adolescent participation in HIV prevention trials. First, sexually active adolescents in this study reported behaviours that put them at similar risk of HIV and other STIs as their adult counterparts. Adolescents in both phases initiated sex earlier than young adult women; had similar numbers of lifetime partners; and similar patterns of sexual concurrency, transactional sexual behaviour and alcohol use. Despite high levels of pregnancy and moderate levels of RTIs/STIs, use of condoms and other contraceptive methods was low or inconsistent for half or more of the participants, regardless of age. Given their sexual risk and barriers to using available risk reduction methods, it is important to determine whether new HIV prevention technologies would be safe, efficacious and acceptable to this population.

Nevertheless, adherence problems identified in two recent trials that aimed to evaluate the efficacy of new vaginal and/or oral HIV prevention methods in African women have raised concern about recruiting young women. Both the FEM-PrEP and the VOICE trials failed to produce evidence of effectiveness for products that were shown to be effective in other populations. Although neither trial recruited women below the age of 18, in the VOICE trial, young, single participants aged 18–24 were much more likely to contract HIV and much less likely to adhere to their study product than married women aged 25 and above [14]. In the FEM-PrEP trial, low adherence was attributed in part to women's low perception of HIV risk [21].

Indeed, the majority of young women – but especially adolescents who participated in our MCT perceived themselves to be at little or no risk of HIV. In that respect, those who joined our clinical study differed from the CFR sample. In particular, young married women and single, out-of-school women appeared particularly vulnerable. Most described sexual partners who were at times controlling or even abusive. They expressed concern about HIV risk, but had difficulty articulating actions they could take on their own to reduce that risk. Despite young married women's apparent need for new, women-initiated HIV prevention methods, the small number of married participants who enrolled in our study suggests additional challenges in recruiting this population.

This paper does not address issues related to the feasibility of adolescent recruitment and retention in HIV prevention trials, or their adherence to products. However, several South African studies examining the feasibility of recruiting adolescents into HIV biomedical and/or vaccine clinical trials found similar rates of incident pregnancy, STIs and HIV among adolescents below the age of 18 and adult women [11, 22]. While adolescents comprised a much smaller proportion of the overall sample in both studies (less than 20%), there were no differences in retention by age groups. A third South African study followed 100 HIV-negative adolescent women and men aged 14–17 over 12 months in preparation for HIV vaccine trials. Follow-up at one year was high (82%), but while incident pregnancy occurred among 7% of female participants, no incident HIV infections were diagnosed [23]. Finally, in a recent analysis of adolescent data from the Carraguard microbicide trial, young women aged 16–17 were no more likely than those aged 18–19 to miss visits, report missed doses or be lost to follow-up. However, they acknowledge that adolescent inclusion in clinical trials may require some modifications to trial design or implementation (i.e. training in youth-friendly counselling and further consideration of informed consent and compensation issues) to support their participation [24].

The study had several limitations. Originally, we aimed to conduct this study in two culturally diverse research sites, with an intended MCT sample size of 300 for subsequent regression analyses: 150 participants each from Pune, India, and Dar es Salaam, Tanzania. Unfortunately, the Indian site was closed after the CFR phase, largely due to difficulties obtaining approval from the local ethics committee (EC) for the MCT. The Indian EC restricted adolescent recruitment to married women only, with husband and/or parental consent required if below the age of 18. The Indian EC's stance further highlights the cultural dimensions that shape young women's sexual risk and their ability or inability to reduce risk by accessing HIV prevention information, products, services – and even clinical trials. Furthermore, observed differences across age groups on those sexual behaviour measures that summarize a participant's entire sexual history have ambiguous interpretations. For example, while older participants were more likely to report “ever” being tested for HIV, this difference might be expected since they are likely to have been sexually active for a longer period of time and also more likely to have become pregnant and tested within the context of antenatal care. And, since we did not collect data on the timing of these behaviours, we are unable to adjust for the unequal periods of risk across the two age groups. However, this limitation does not apply to the reported number of sex partners per year, questions relating to the first sexual experience, or to measures that refer to a behaviour within a specified time frame (e.g. sex in the past week or incident pregnancy). Finally, while the qualitative data from our CFR participants provide a richer understanding about the contexts within which young women experience risk, these data cannot be linked directly to MCT participants – who were recruited from the same venues, but through different approaches.

## Conclusions

Adolescents under age 18 are at risk of negative SRH outcomes, including pregnancy, STIs, RTIs and HIV. They are less likely than their older counterparts to report accessing HIV testing, obtaining gynaecological exams or using protective technologies such as contraception. With adolescents at similar, if not higher risk, than their young adult counterparts, combined with their underuse of current preventive services, their inclusion in clinical trials for new preventative technologies is clearly warranted – but additional support for recruitment, retention and product adherence may be required. Their access to new technologies such as vaginal microbicides or PrEP may be compromised unless research is undertaken to assess the safety, acceptability and effectiveness of these products in this age group. Adolescent exclusion from HIV prevention trials may hinder access to new HIV prevention technologies, once available.
